# Ultrasound hyperthermia induces apoptosis in head and neck squamous cell carcinoma: An *in vitro* study

**DOI:** 10.4317/medoral.21245

**Published:** 2017-04-08

**Authors:** Guoxin Ren, Houyu Jv, Zhuowei Tian, Shalva R. Gvetadze, Jingzhou Hu, Mei Zhao, Ge Zhou, Chenping Zhang, Zhiyuan Zhang

**Affiliations:** 1 MD, DDS, PhD. DDS. Department of Oral and Maxillofacial-Head and Neck Oncology, Ninth People’s Hospital, Shanghai Jiao Tong University School of Medicine, Shanghai Key Laboratory of Stomatology and Shanghai Research Institute of Stomatology, China; 2 MD, DDS. Central Research Institute of Dentistry and Maxillofacial Surgery, Congenital Maxillofacial Defects and Deformations, Moscow, Russia; 3 MD, PhD. Department of Head and Neck Surgery, The University of Texas MD Anderson Cancer Center, Houston, Texas 77030, USA

## Abstract

**Background:**

Hyperthermia is considered an efficient complement in the treatment of head and neck squamous cell carcinoma (HNSCC). Hyperthermia induces cell apoptosis in a temperature- and time-dependent manner. However, the molecular mechanism of hyperthermia remains unclear. The aim of this study was to investigate the mechanism of apoptosis induced by ultrasound hyperthermia in HNSCC cell lines HN-30 and HN-13.

**Material and Methods:**

We examined the dynamic changes of early apoptosis and secondary necrosis in HN-30 and HN-13 cells treated by hyperthermia at 42°C for 10 min. We further examined mitochondrial membrane potential *in vitro* by ultrasound hyperthermia for different heating temperatures (38-44°C, 10 min) and heating times (42°C, 10-50 min). After heating by ultrasound at 42°C for 10 min, the apoptosis index achieved its highest level at 8 h after treatment, decreased rapidly and remained constant at a reduced level at 12 h.

**Results:**

The level of secondary necrosis increased with the level of early apoptosis but remained at a higher level until 14 h. The level of secondary necrosis correlated with the level of early apoptosis (HN-13: r=0.7523, *P*=0.0030; HN-30: r=0.6510, *P*=0.016). The fractions of cells with low mitochondrial membrane potential (Δψ) in the heating-temperature grads group and heating-time grads group decreased significantly over time. Therefore, HN-30 and HN-13 cells developed apoptosis after ultrasound hyperthermia treatment with decreases in the mitochondrial transmembrane potential level.

**Conclusions:**

Ultrasound hyperthermia induces apoptosis in HN-30 and HN-13 cells, possibly via the mitochondrial caspase pathway.

** Key words:**Head and neck squamous cell carcinoma, apoptosis, mitochondrial membrane potential, ultrasound hyperthermia.

## Introduction

Head and neck cancer accounts for 4% of all malignancies worldwide and 5% of all cancer fatalities (1). Conventional treatment methods, such as ablation, chemotherapy and radiotherapy, cannot be used individually to treat head and neck squamous cell carcinoma (HNSCC). Instead, a combined treatment method is necessary.

Hyperthermia cancer therapy is currently used either for ablation purposes as an alternative to surgery or in combination with chemotherapy and/or radiation therapy to enhance the effects of those traditional therapies. Hyperthermia cancer therapy kills tumor cells directly and/or sensitizes the cells to chemotherapy or radiation therapy (2,3). Hyperthermia is relatively selective to tumor tissue given the altered anatomical/physiological features of tumor tissue (e.g., the presence of leaky vasculature, hypoxia, acidosis, poor lymphatic drainage and increased interstitial pressure) compared with normal tissue (4). Studies have demonstrated that hyperthermia treatment induced by electromagnetic inhibits proliferation and induces apoptosis in tumor cells (5). More recent studies have demonstrated an increase in immunological attacks against tumors after hyperthermia therapy induced by electromagnetic, which was believed to be achieved through the activation of heat-shock proteins (HSPs) and the subsequent modulation of the innate and adaptive immune responses against tumor cells (6,7).

Mechanisms to induce hyperthermia include thermal conduction using a circulating liquid and exposure by electromagnetic (radiofrequency, microwaves or infrared) or acoustic waves (ultrasound). In radiative electromagnetic and ultrasound hyperthermia, wave interference is used to heat deep target regions and focus the heat on a predefined target volume (8). Ultrasound technology has significant advantages due to its greater penetration, satisfactory temperature control and acute temperature measurement. Therefore, ultrasound thermal therapy is considered a promising thermotherapy technique. The mechanism underlying the anti-tumor effect of ultrasound thermal therapy remains unclear. In this work, we investigated the early morphological changes of tumor cells induced by ultrasound hyperthermia. We studied the characteristics of early apoptosis and secondary necrosis in HNSCC cell lines HN-30 and HN-13. In addition, we measured the mitochondrial membrane potential to elucidate the likely role of mitochondrial membrane permeabilization (MMP) in apoptosis induced by ultrasound hyperthermia.

## Material and Methods

**Figure 1:**
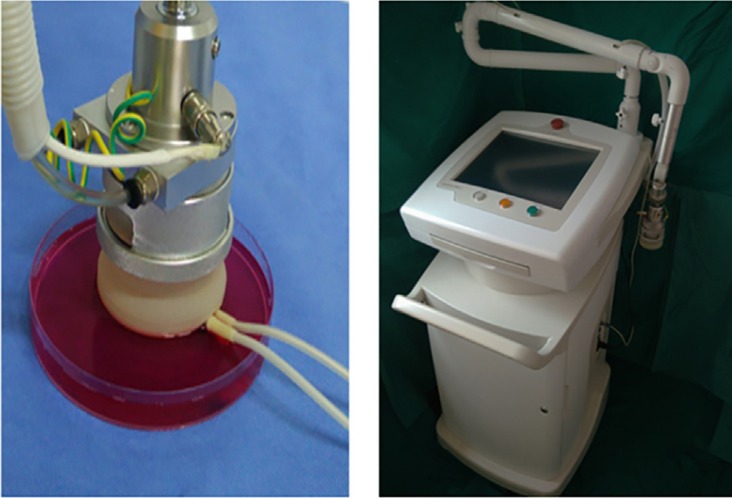
Ultrasonic thermal therapy system.

- Reagents. Acridine orange (AO) and ethidium bromide (EB) were obtained from Gibco (Carlsbad, CA, USA). An Annexin V-FITC Apoptosis Detection Kit and Mitochondrial Membrane Potential Detection Kit were purchased from BD Biosciences (San Jose, CA, USA).

- Materials. An ultrasonic thermal therapy system (Fig. 1) was developed by the Institute of Biomedical Instrument of Shanghai Jiao Tong University. Briefly, ultrasound was generated by four square transducers (PZT-5) with dimensions of 32×32 mm. The system had two resonant frequencies (1 and 3.5 MHz) for the different treatment objects. The power signal feeding the transducers was generated by a frequency generator and amplified by a LC syntonic circuit (an electric circuit consisting of an inductor, represented by the letter L, and a capacitor, represented by the letter C, connected together, act as an electrical resonator, storing energy oscillating at the circuit’s resonant frequency and amplify the power signal). The electrical impedance of the transducer was matched to the output impedance of the amplifier by the LC matching network. A needle sensor was used to measure the actual temperature of the treatment target so that the power of the ultrasound transducers could be accurately controlled. Movement of the treatment head in the x, y or z direction was performed by a three-dimensional (3D) movement machine-compatible mechanical system. A plastic membrane covered the treatment head such that the liquid could be infused into the head. An ultrasound hyperthermia system, including the target location, ultrasound dose, frequency and temperature control, could be implemented by the supporting software with the participation of the investigators (9). In this work, the frequency, voltage and power of the system were 1 MHz, 33 V and 25 W, respectively.

- Cell culture. Human HNSCC cell lines HN-13 and HN-30 were kindly provided by the University of Maryland Dental School. These cell lines were cultured in DMEM supplemented with 10% heat-inactivated FBS (GIBCO BRL, NY, USA), penicillin (100 units/ml) and streptomycin (100 μg/ml) at 37°C in a humidified 5% CO2 atmosphere. HN-13 and HN-30 cell suspensions (200 ml) were harvested and headed *in vitro* by the ultrasound hyperthermia system.

Acridine orange (AO) and ethidium bromide (EB) staining for cell apoptosis morphology. AO/EB staining of the HN-30 and HN-13 cells was performed to confirm the ultrasound hyperthermia-induced apoptosis pattern. HN-30 and HN-13 cells were treated by the ultrasonic thermal therapy system for 10 min at 42°C. Cells (1×105/200 μl) were harvested after being cultured for 1 h at 37°C in a humidified 5% CO2 atmosphere and washed twice with phosphate-buffered saline (PBS). The cells were then incubated in the dark for 15 min with a 500 μg/ml AO/EB mixture solution at room temperature and immediately observed under a fluorescence microscope (Nikon, Tokyo, Japan).

Flow cytometry by Annexin V-propidium iodide (PI) staining. An Annexin V-FITC Apoptosis Detection Kit (BD biosciences, USA) was used to estimate the early apoptosis and secondary necrosis of HN-30 and HN-13 cells. HN-30 and HN-13 cells were treated by the ultrasonic thermal therapy system for 10 min at 42°C. Cells (1×106/500 μl) were harvested after being cultured for 1, 2, 3, 4, 5, 6, 7, 8, 10, 12, 14, and 16 h at 37°C in a humidified 5% CO2 atmosphere. Then, the cells were washed twice with cold PBS and re-suspended in 400 μl of Annexin V binding buffer. The cells were double-labeled with 5 μl of Annexin V-fluorescein and 5 μl of PI and incubated at room temperature in the dark. After 15 min of incubation, the cells were analyzed by a FACScan flow cytometer. Data analysis was performed with the standard Cell Quest software (Beckman Coulter Inc, Brea, CA,USA). All experiments were performed in duplicate, and reproducibility was assessed in three independent experiments.

Measurement of the mitochondrial membrane potential. A Mitochondrial Membrane Potential Detection Kit (BD Biosciences, USA) was used to estimate the early apoptosis of HN-30 and HN-13 cells. HN-30 and HN-13 cells were treated by the ultrasonic thermal therapy system as detailed in  Table 1, Table 2. The cells (1×106/500 μl) were harvested after being cultured for 6 h at 37°C in a humidified 5% CO2 atmosphere. Then, the cells were labeled with 5 μl of JC-1 working solution and incubated at 37°C in a CO2 incubator. After 15 min of incubation, the cells were washed twice with assay buffer. The cells were gently re-suspended in 0.5 ml of assay buffer and analyzed by a FACScan flow cytometer. Data analysis was performed with the standard Cell Quest software. All experiments were performed in duplicate, and reproducibility was assessed in three independent experiments.

**Table 1:**
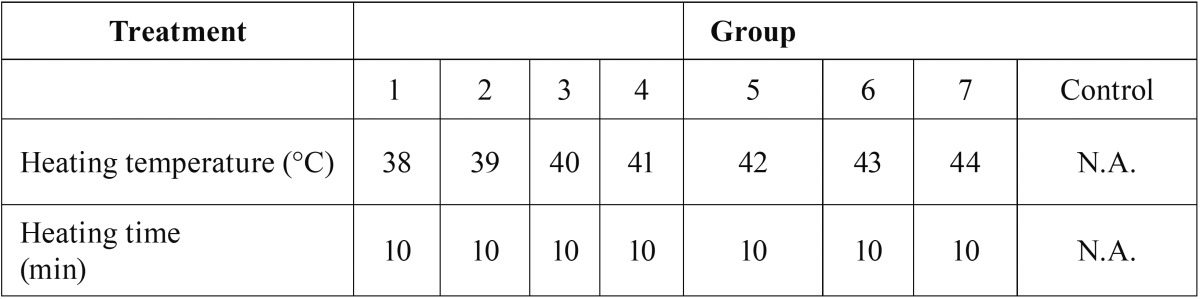
Heating temperature and heating time of HN-30 and HN-13 cells treated by ultrasound hyperthermia at different heating temperatures.

**Table 2:**
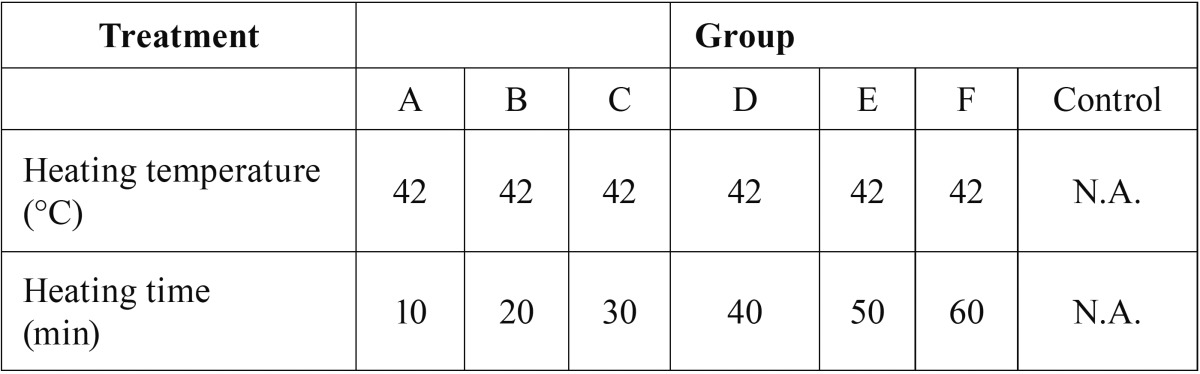
Heating temperature and heating time of HN-30 and HN-13 cells treated by ultrasound hyperthermia for different heating times.

Statistical analysis. Data are represented as the mean ± standard deviation (SD) of more than three independent experiments. Statistical analysis was performed using Student’s t test, and *P*-values less than 0.05 were considered significant.

Ethical approval and Informed consent. Ethical approval was granted by Institutional Clinical Research Supervision Committee and Informed consent was not necessary.

## Results

**Figure 2:**
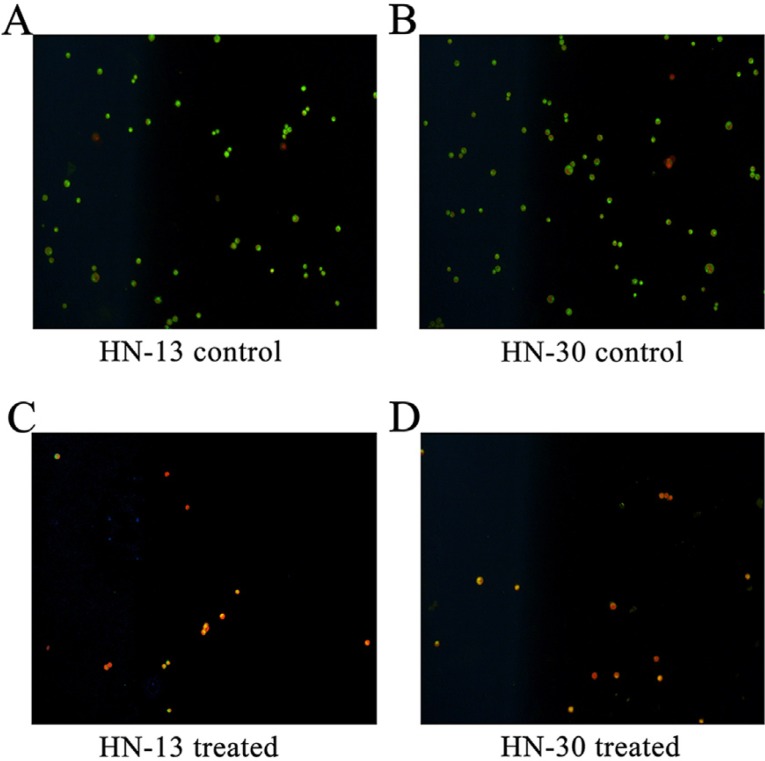
AO/EB staining of normal HN-13 (A) and HN-30 (B) cells, where the live cells emit green fluorescence under the fluorescence microscope (×100). AO/EB staining of HN-13 (C) and HN-30 (D) cells after 1 h of 42°C ultrasound hyperthermia for 10 min. The majority of cells exhibited orange fluorescence, indicating that their cell membranes were injured by ultrasound hyperthermia (×100). Similar results were obtained from experiments performed in triplicate.

Ultrasound hyperthermia kills HNSCC cells. In order to test the likely anti-tumor role of ultrasound hyperthermia on HNSCC cells, we first examined the impact of ultrasound hyperthermia on HN-30 and HN-31 cells by AO/EB staining. AO can pass through the cell membrane and stain the DNA of the cell nucleus green, whereas EB is only able to penetrate the injured cell membrane and stain the DNA of the cell nucleus orange (10). Our AO/EB staining showed that 1 h after treated by 42°C ultrasound hyperthermia for 10 min, most cells exhibited orange fluorescence, indicating that their cell membranes were injured by the ultrasound hyperthermia. Under ultrasound hyperthermia, live cells glowed bright fluorescent green, whereas cells glowed fluorescent orange because the dyes passed through the injured cell membrane. (Fig. 2) These preliminary results indicate that the ultrasound hyperthermia system was able to kill HNSCC cells.

**Figure 3:**
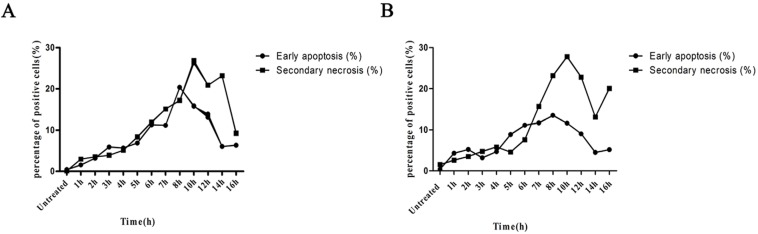
The curves of the changes of early apoptosis and secondary necrosis in HN-13 and HN-30 cells treated at 42°C for 10 min, as we shown in the table 3. Both apoptosis and necrosis curves reach a peak and apoptosis curves decrease rapidly, while necrosis curves remaining at a high level for a certain period.

**Figure 4:**
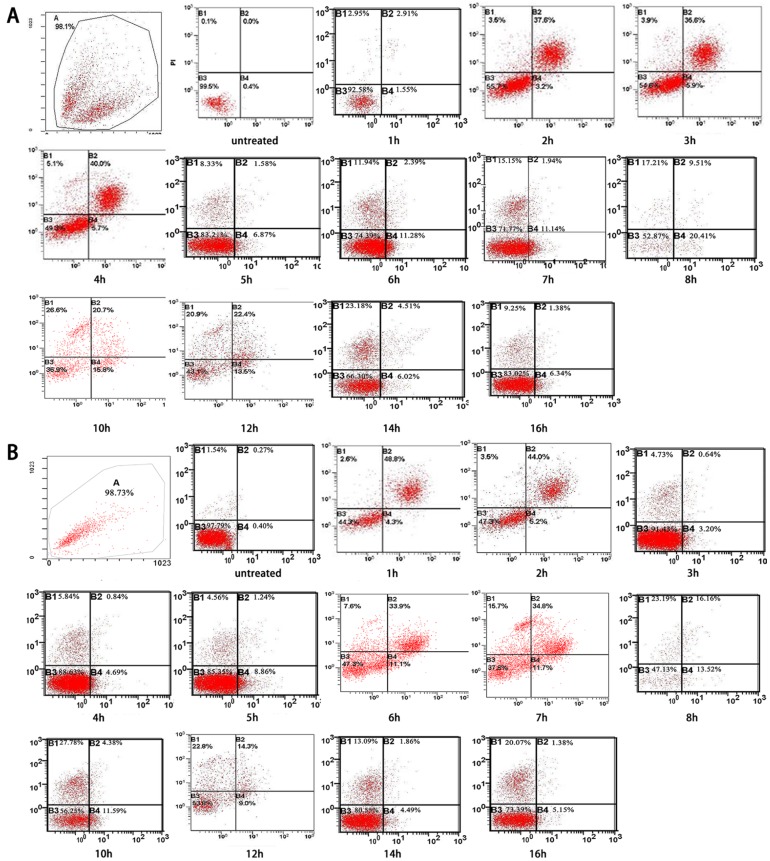
The representative FACS analysis of the results summarized in table 3, showing the gating and labelling the different populations (live, early apoptotic, necrotic) in HN-13 and HN-30 cells for each experimental condition.

Ultrasound hyperthermia induces both Apoptosis and necrosis In HNSCC cells. Early apoptosis and secondary necrosis were detected by annexin V PI staining in HN-30 and HN-13 cells at 1 h after treated by 42°C ultrasound hyperthermia for 10 min. ( Table 3) demonstrates that cells that exhibited early apoptosis after 1 h achieved a peak value between 6 and 8 h, then decreased rapidly until 12 h and finally remained at a constant low level (Figs. 3,4). Secondary necrosis exhibited only slight differences, remaining at a high level for a certain period after achieving a peak value between 6 and 8 h. Secondary necrosis was positively correlated with the rate of early apoptosis (HN-13: r=0.7523, *P*=0.0030; HN-30: r=0.6510, *P*=0.016).

**Table 3:**
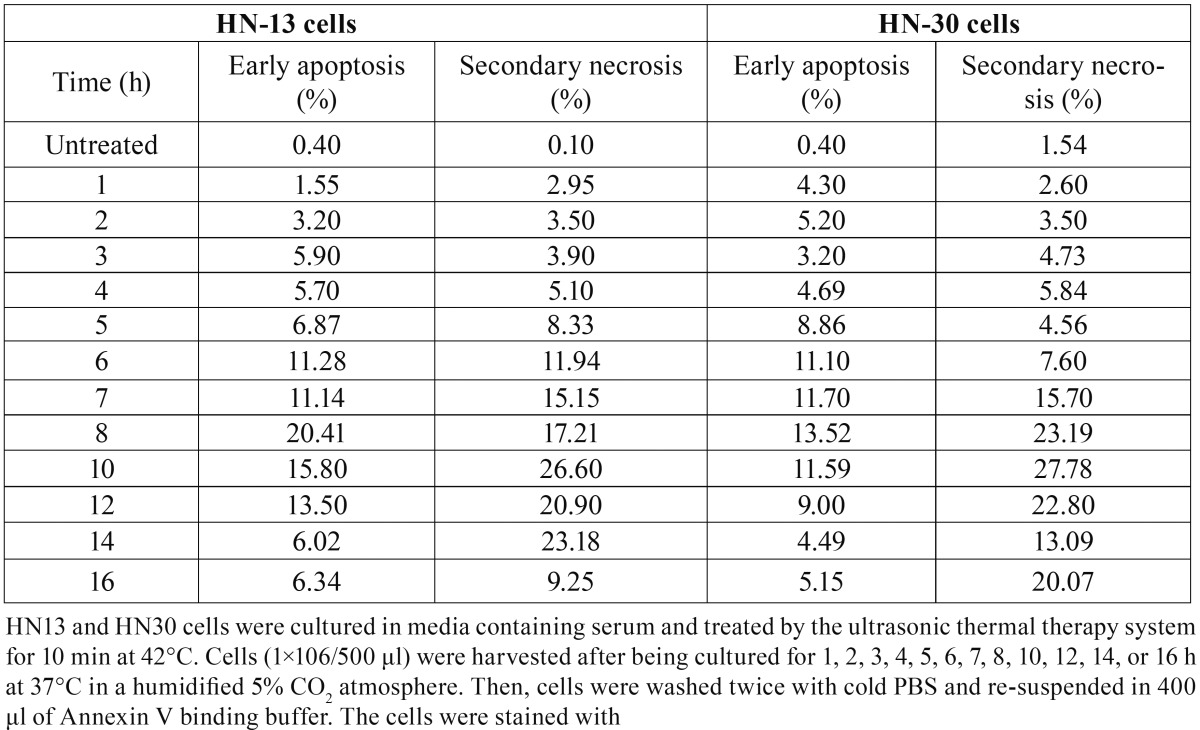
Dynamic changes of early apoptosis and secondary necrosis in HN-13 / HN-30 cells treated at 42°C for 10 min (%).

**Figure 5:**
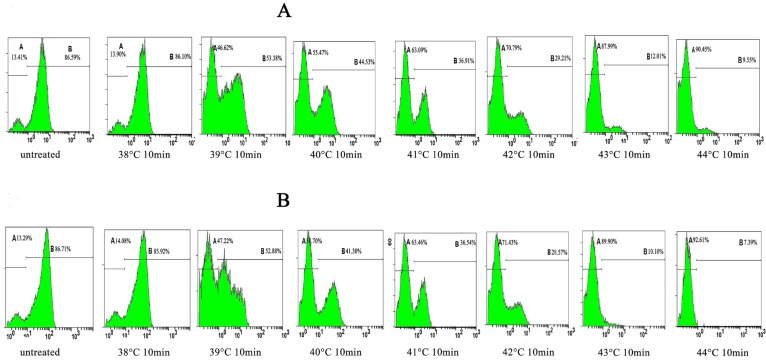
The representative FACS analysis of the results summarized in Table 4, showing the monomeric JC-1 (green fluorescence intensity) levels of in HN-13 and HN-30 cells for each experimental condition.

**Figure 6:**
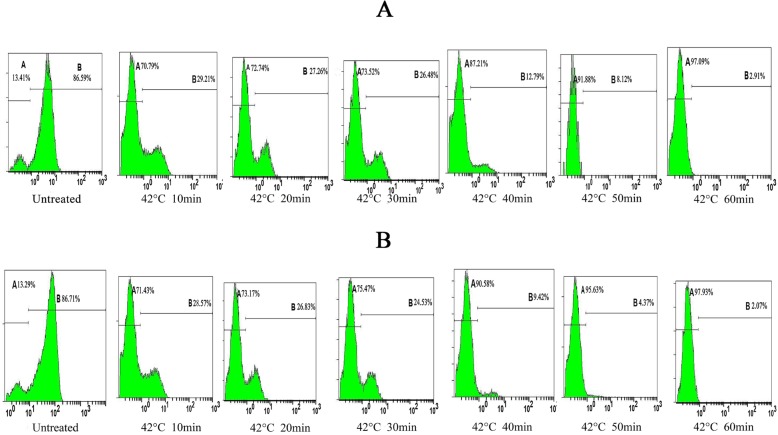
The representative FACS analysis of the results summarized in Table 5, showing the monomeric JC-1 (green fluorescence intensity) levels of in HN-13 and HN-30 cells for each experimental condition.

Ultrasound hyperthermia disrupts the mitochondrial membrane potential of HNSCC cells. The level of the mitochondrial transmembrane potential (Δψ) with variations in the heating temperature and heating time was shown in tables  Table 4, Table 5, in which while heating at 38°C caused only a slight effect on Δψ, (*P*>0.05), heating between 39°C and 44°C decreased Δψ considerably (*P*<0.05), especially, Δψ decreased considerably after 1 h of ultrasound hyperthermia at 42°C for 10 min and continued to decrease with further heating (Figs. 5,6). Together, our results show that ultrasound hyperthermia is able to disrupt the mitochondrial membrane potential of HNSCC cells.

**Table 4:**
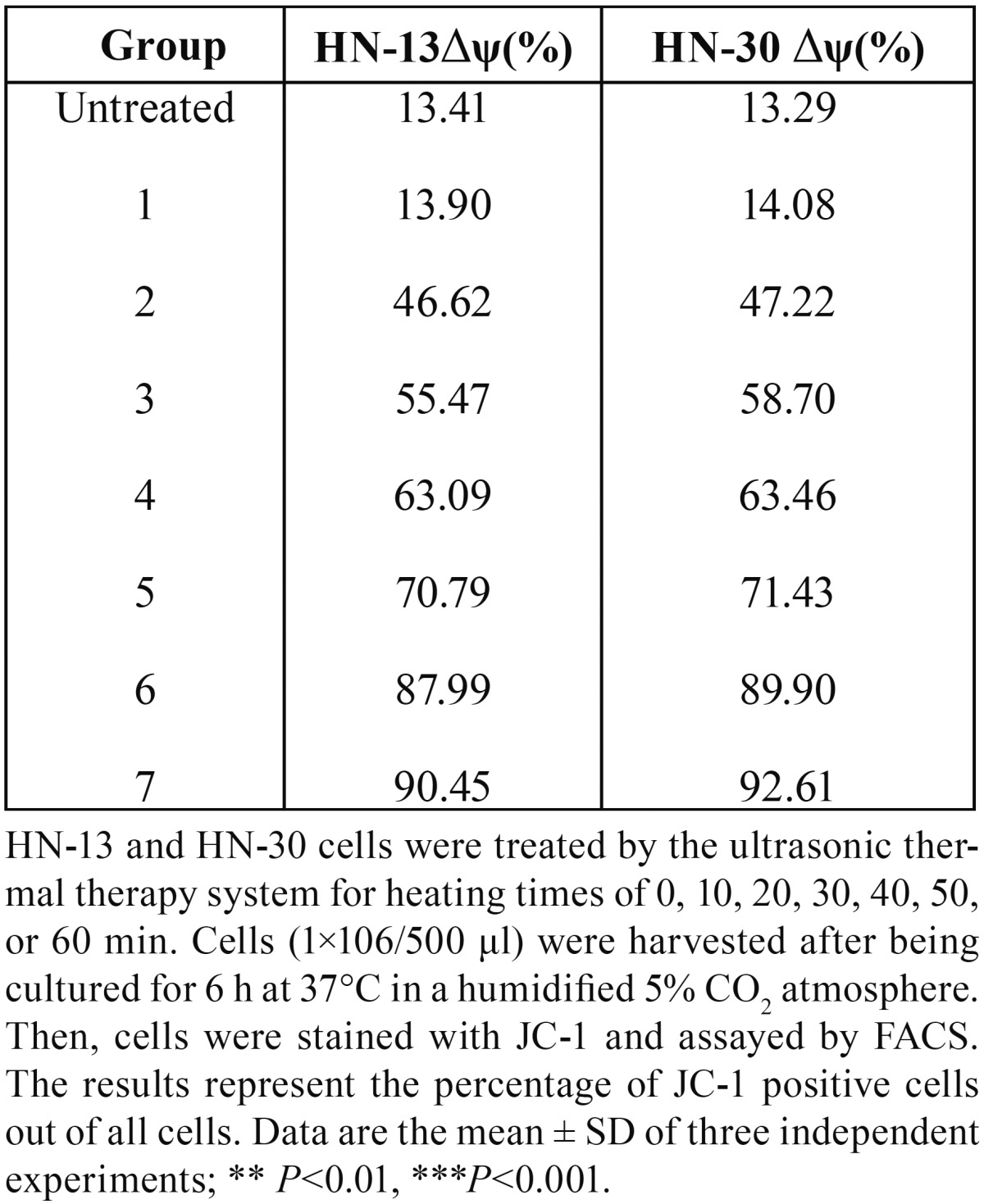
Changes in the Δψ of HN-13 cells treated at different heating temperatures.

**Table 5:**
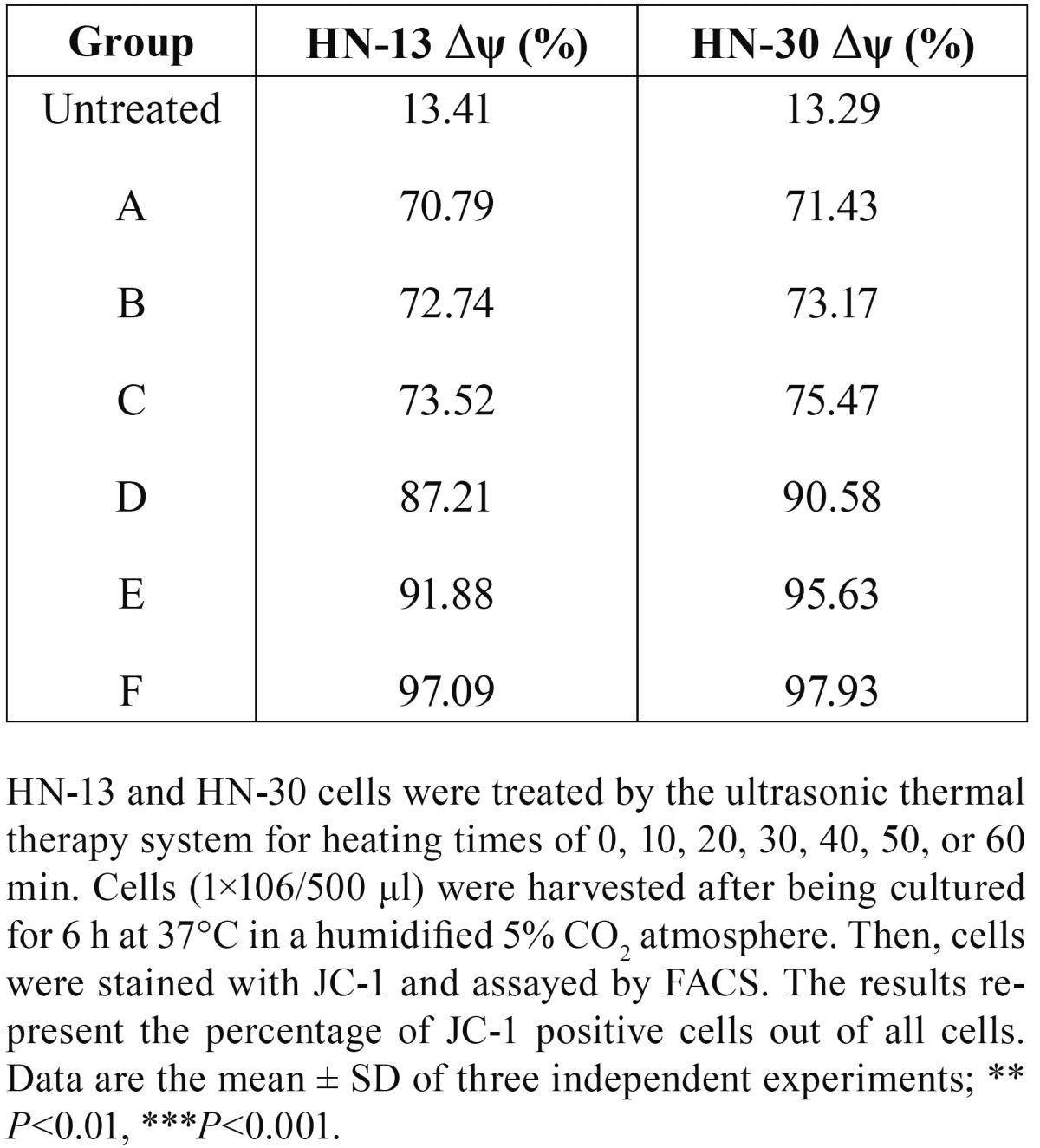
Changes in the Δψ of HN-13 cells treated for different heating times.

## Discussion

Our research suggests that ultrasound hyperthermia has a significant influences on HNSCC viability and the mitochondrial transmembrane potential (Δψ) in a dose- and time-dependent manner.

Apoptotic and necrotic cells exhibit characteristic early morphological differences in cell membrane integrity. An AO/EB mixture stain was used because AO can pass through the cell membrane and stain the DNA of the cell nucleus green, whereas EB is only able to penetrate the injured cell membrane and stain the DNA of the cell nucleus orange (10). The AO/EB stain enabled us to observe destruction of the membrane of HN-30 and HN-13 cells upon ultrasound hyperthermia treatment. Apoptosis and some forms of necrosis share a common pathway in the early stage; however, the intracellular energy levels and mitochondrial function are rapidly compromised in necrosis but not in apoptosis (11). Cells undergo apoptosis under the normal ATP supply and oxidative stress; otherwise, they undergo necrosis.

Annexin V-PI staining is a method with a strong specificity for apoptosis and necrosis in cells. Annexin V binds to phosphatidylserine (PS), which externalizes at early-stage apoptosis. In contrast, PI is unable to permeate live and apoptotic cells but stains dead cells with red fluorescence by binding tightly to the nucleic acids in the cell. Our observations in HN-30 and HN-13 cells indicated that the cells exhibited early apoptosis at 1 h after treated by 42°C ultrasound hyperthermia for 10 min. A peak value was achieved between 6 and 8 h and decreased rapidly. However, a less obvious trends for secondary necrosis was noted in HN-30 and HN-13 cells; necrosis remained at a high level for a certain period after achieving a peak value in conjunction with the apoptosis rate between 6 and 8 h. Secondary necrosis was positively correlated with the rate of early apoptosis in cells. Given that cell membrane integrity can further degrade during late apoptosis, part of late apoptotic cells were sorted as secondary necrotic cells because PI enters the cytoplasm and Annexin V-PI staining indicated doubly labeled positive cells.

Several oncoproteins, tumor suppressor gene products, viral virulence factors and pharmacological agents modulate apoptosis via direct effects on mitochondria, and mitochondrial transmembrane potential (MMP) constitutes an essential step of the intrinsic pathway leading to apoptosis (12). The most distinctive marker of MMP is the dissipation of the mitochondrial transmembrane potential (Δψ). Δψ results from the asymmetric distribution of protons and other ions on both sides of the inner mitochondrial membrane, thus creating a chemical (pH) and electric gradient that is essential for mitochondrial function (13). Cells undergoing apoptosis exhibit a decreased Δψ (14). Δψ disruption occurs before cells exhibit nuclear DNA fragmentation or aberrant exposure of PS on the outer cell membrane leaflet, indicating that it constitutes an early common event of the apoptotic cascade (15). In our study, Δψ decreased during 42°C ultrasound hyperthermia treatment for 10 min and remained at a low level in the cells undergoing 42°C ultrasound hyperthermia treatment for 20 to 50 min, exhibiting only slight variations. Furthermore, Δψ decreased significantly in the groups undergoing 39 to 44°C ultrasound hyperthermia for 10 min (Tables  Table 4, Table 5). The maintenance of Δψ relies on the stability of the mitochondrial permeability transition (MPT) pore structure. The factors that induce apoptosis could open the MPT pore, thus causing cytochrome c to be released from the mitochondria and forming the apoptosome complex, which includes apoptosis protease activating factor-1 (APAF-1), procaspase 9, and ATP (or the less abundant dATP) (16). In an ATP or dATP-dependent manner, procaspase 9 is activated to caspase 9, which then activates caspase 3 to initiate the final executioner phase of apoptosis (17). Our research suggests that the two apoptosis-related indices (heating temperature and heating time) had significant influences on Δψ. A heating temperature of over 39°C or 42°C treatment for greater than 10 min initiated the apoptosis pathway by markedly decreasing Δψ.

In accordance with previous reports (18), this study demonstrates that ultrasound hyperthermia destroys the structure of the mitochondrial membrane to induce apoptosis in HN-30 and HN-13 cells. However, the mechanism by which this effect is mediated has not been identified. Both APAF-1 and procaspase 9 are downstream substrates of cytochrome c. Procaspase 9 phosphorylates and activates CASPASE 3, which initiates the final executioner phase of apoptosis. Further investigation is required to determine whether the apoptosome complex function is hyperactivated by ultrasound hyperthermia and whether increased apoptosome complex function affects CASPASE 3 expression.

In summary, this study reports that HN-30 and HN-13 cells developed apoptosis after heating by ultrasound hyperthermia in conjunction with decreases in Δψ. Ultrasound hyperthermia induces apoptosis in HN-30 and HN-13 cells, possibly via the mitochondrial caspase pathway. The experiments presented here suggest that ultrasound hyperthermia may efficiently treat HNSCC by inducing apoptosis.
